# Isolated fracture of the humeral trochlea: a case report and review of the literature

**DOI:** 10.1186/s13256-015-0564-1

**Published:** 2015-05-28

**Authors:** Najib Abbassi, Najib Abdeljaouad, Abdelkrim Daoudi, Hicham Yacoubi

**Affiliations:** 1 Hay Essalam Lot Laalej Res Ennour N709, Oujda, Morocco; 2 Department of Orthopedic Surgery and Traumatology, University Hospital Med VI, Oujda, Morocco

**Keywords:** Humerus, Isolated fracture, Osteosynthesis, Trochlea

## Abstract

**Introduction:**

Isolated fracture of the trochlea is a rarely reported entity. To the best of our knowledge, only 15 cases have been published. We report the case of a patient with an isolated fracture of the trochlea and discuss through a literature review the underlying mechanisms and the clinical, radiological and therapeutic features of this lesion. This work will significantly advance our understanding of this particular fracture.

**Case presentation:**

A 21-year-old Caucasian man received an elbow injury. An anteroposterior radiograph showed only an irregularity of the medial joint space, but a lateral radiograph showed an intra-articular half-moon-shaped fragment that had moved up and forward. A computed tomography scan confirmed an isolated fracture of his trochlea. Open reduction and internal fixation were performed with a good outcome.

**Conclusion:**

An isolated fracture of the trochlea is rare. The mechanisms generating this fracture are complex. As with other front-line fractures of the distal end of the humerus, such as the capitulum, we recommend open reduction and internal fixation for displaced fractures, with excision of the small osteochondral fragments that do not permit any osteosynthetic repair.

## Introduction

The first description of an isolated fracture of the humeral trochlea was in 1853 by Laugier. Thus, the trochlea fracture is also sometimes known as Laugier’s fracture [1]. Although this description is old, these fractures remain rare. Our review of the literature identified only eight reported cases [2-9] and two limited series relating respectively to two and five cases [10,11]. We report a case of an isolated fracture of the trochlea that was treated surgically. The purpose of this study is to discuss the mechanisms and the diagnostic and therapeutic issues relating to this entity.

## Case presentation

**Figure 1:**
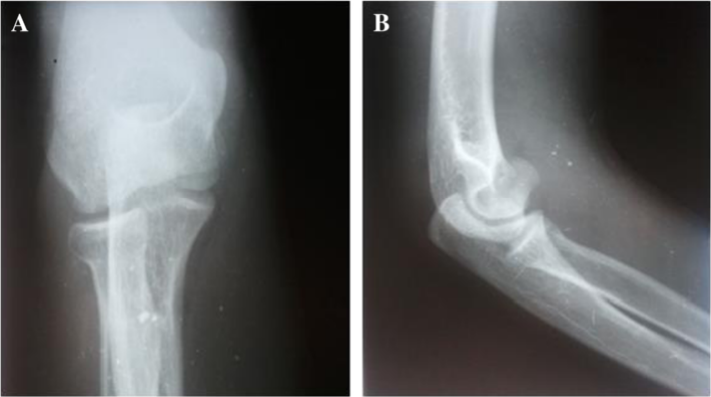
Initial radiograph. **(A)** Anteroposterior view and **(B)** lateral view.

**Figure 2:**
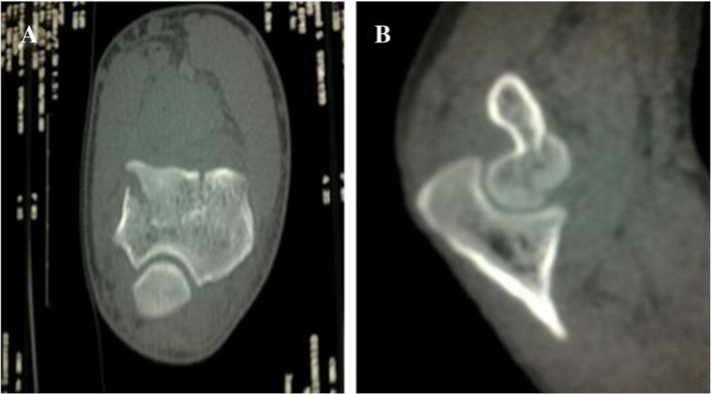
Computed tomography scan. **(A)** Axial view and **(B)** sagittal view.

A 21-year-old Caucasian man had a fall, landing on the palm of his right hand with his elbow extended and supinated. A clinical examination found pain and swelling in the medial part of his elbow, with restricted flexion-extension but pronation and supination were free. An anteroposterior radiograph showed only an irregularity of his medial joint space, but a lateral radiograph showed an intra-articular half-moon-shaped fragment that had moved up and forward, making us suspect a capitellar fracture (Figure 1). A computed tomography (CT) scan confirmed an isolated front-line fracture of his trochlea, without participation of the capitellum (Figure 2).

Open reduction and internal fixation was planned for our patient. The joint was opened through a medial approach, passing between the triceps brachii in the back and brachialis in the front. His ulnar nerve was dissected and protected. Accessing the joint capsule required disinsertion of the humeral part of his pronator teres, without interrupting the medial collateral ligament, which was intact. His trochlea was fractured across the front line, with persistence of the posterior wall. The fragment had moved up and forward without any compaction or loss of cartilage substance.

**Figure 3:**
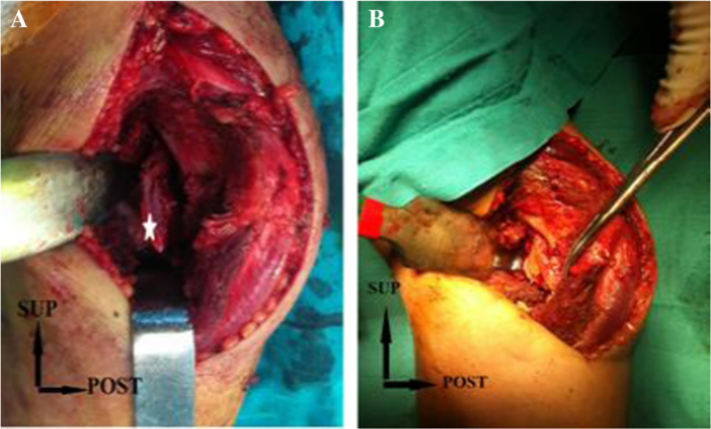
Fracture of the trochlea. **(A)** Before reduction; the fracture is marked by five-point star. **(B)** After reduction and fixation. SUP, superior; POST, posterior.

**Figure 4:**
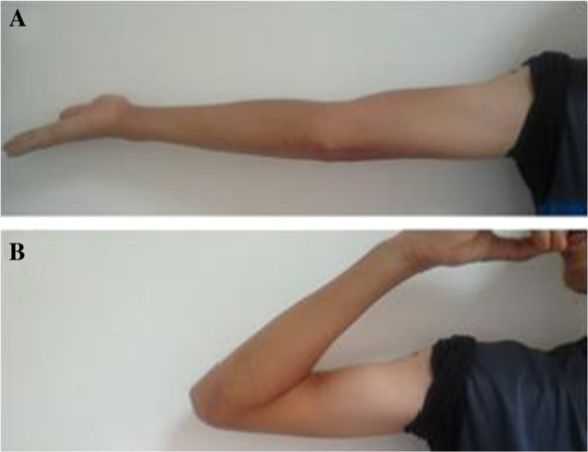
Recovery of **(A)** extension and **(B)** flexion.

**Figure 5:**
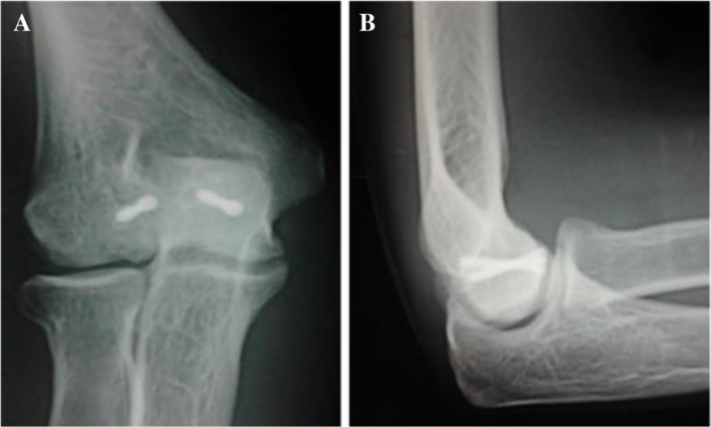
Radiograph after two years of follow-up. **(A)** Anteroposterior view and **(B)** lateral view.

The reduction was facilitated by bending his elbow to retract the brachii forward. The fixation was obtained by inserting two Herbert screws of 20mm length into his cartilage (Figure 3). His elbow was immobilized in a back splint in a flexed position for 15 days to allow soft tissue healing and minimize pain for our patient. Rehabilitation of his elbow was based on active assisted mobilization for three months. After two years of follow-up, his elbow was stable and painless with a normal range of motion (Figure 4). Radiological imaging showed no signs of trochlear osteonecrosis or osteoarthritis (Figure 5).

## Discussion

Fracture of the trochlea is usually associated with elbow dislocation and capitellar or medial condylar fracture [12]. Isolated fracture of the humeral trochlea is very rare. This is explained by its position deep within the trochlear notch cavity and the absence of any muscular or ligamentous attachments at this level, which protects it against direct and indirect trauma [13]. Furthermore, the ulno-humeral joint is subjected to very light compressive and shear forces compared to those experienced by the radio-humeral joint, which explains the high frequency of capitellar fractures compared to trochlear fractures [14]. Trochlear fractures reported in the literature were the result of both high-energy (for example, a road traffic accident) and low-energy (falls) traumas. The position of the elbow was variable, bent in some cases, extended in others (Table 1).

**Table 1:** **Energy and mechanism of trauma**

**Energy and mechanism**	**Number of cases**	**References**
**High energy of trauma**	9	[2-4,7,11]
**Low energy of trauma**	6	[5,6,8-10]
**Elbow in bending position**	7	[10,11]
**Elbow in extended position**	2	[3,4]

Table 1 shows that an isolated fracture of the humeral trochlea has no specific characteristics. Tetsuya *et al.* emphasized the role of varus stress, which displaces the compressive forces of the radio-humeral compartment to the ulno-humeral compartment [3]. Evaluation of radiographs in the anteroposterior view may show an irregularity at the ulno-humeral joint [2,3,5-7], but the image can be interpreted ‘normal’ [4,8-11]. In a lateral view, the appearance of an articular half-moon-shaped fragment moved up and forward could suggest a capitellar fracture. For this reason, diagnosis is based on the results of a CT scan [2,3,6,8,10,11]. CT allows the treating physician to determine the size of the fragment and its displacement, thus guiding the surgical procedure. All cases reported in the literature were treated by open reduction and internal fixation. The medial approach was the most used; the anterior approach was used only twice (Table 2). Both approaches provide good exposure of the fracture and the choice depends on the surgeon’s preference. We noted a discrepancy between the authors in the choice of screws, the seat of fixation and the direction of screws. A Herbert screw was used in six cases. A cancellous screw with head was used in seven cases. Kirschner wires (K-wires) were used in two cases, because of the small size of the fragments. The approach and the means of osteosynthesis are summarized in Table 2.

**Table 2:** **Approach and the means of osteosynthesis**

**Approach**	**Number of cases**	**References**
**Medial approach**	13	[2,4,5,7-11]
**Anterior approach**	2	[3,6]
**Herbert screw**	6	[3,5,6,8,11]
**Cancellous screw**	7	[2,4,7,9,10]
**Kirschner wires**	2	[10]

In the reported cases, the cancellous screws were passed from the non-articular area. Their direction was oblique, from front to back and from medial to lateral, fixing the trochlea to the capitulum. Alternatively, Herbert screws were inserted into the articular surface buried beneath the cartilage; their direction was perpendicular to the fracture line, securing the fragment of the trochlea to the posterior wall with maximum compression. We opted for this type of osteosynthesis because it is more stable from a biomechanical point of view. Patients whose fractures were treated with screws were immobilized for one to two weeks. K-wires were used in only two cases, and the patients were immobilized for three weeks. Fracture healing was obtained in all cases within six weeks. All results were good, except for one patient whose fracture was treated by K-wire, who developed osteoarthritis after three years of follow-up [10].

## Conclusion

Isolated fracture of the trochlea is rare. The mechanisms generating this fracture are complex. We recommend open reduction and internal fixation for displaced fractures, with excision of the small osteochondral fragments that do not permit any osteosynthetic repair.

## Consent

Written informed consent was obtained from the patient for publication of this case report and any accompanying images. A copy of the written consent is available for review by the Editor-in-Chief of this journal.
